# Health literacy: relationship with the family caregiver role

**DOI:** 10.1515/med-2026-1449

**Published:** 2026-06-01

**Authors:** Juliana Sofia Ortiga Nogueira, Carla Alexandra Silva Pombo Soares, Nadirlene Pereira Gomes, Amâncio António de Sousa Carvalho

**Affiliations:** Health School, University of Trás-os-Montes and Alto Douro, Vila Real, Portugal; Nursing School, Federal University of Bahia, Salvador, Bahia State, Brazil; Hospital da Luz, Vila Real, Portugal; Local Health Unit of Trás-os-Montes and Alto Douro, Douro Community Care Unit, Rua Dr. José de Sousa Pereira, Peso da Régua, Portugal

**Keywords:** family caregivers, health literacy, self-efficacy, family health, family nursing

## Abstract

**Objectives:**

Health literacy (HL) is a public health challenge. The family caregiver (FC) needs to adhere to the caregiver role to perform this role with good self-care performance. To analyze the relationship between the HL of FC and the perception of self-efficacy (SE) in behaviors of adherence to the role of FC.

**Methods:**

This is a descriptive-correlational and cross-sectional study, in which 111 FC participated, users of a Portuguese Family Health Unit. We used a telephone interview questionnaire to collect data, which was processed using SPSS.

**Results:**

Of the total sample (n=111), the majority of FC fell into the problematic level (47.7 %) of General HL. The mean ranking of the HL categories differed significantly in the behaviors of adherence to the FC role “Encouraging the Dependent Family Member (DFM) independence” (MW: p=0.033), “Promoting the use of appropriate clothing for the DFM” (MW: p=0.001) and “Assisting the DFM in his/her self-surveillance” (MW: p=0.000), with FC with a higher level of HL having a higher SE.

**Conclusions:**

General HL relates to encouraging independence, using appropriate clothing and assisting the DFM in self-monitoring. This diagnosis constitutes an important tool for implementing appropriate interventions.

## Introduction

The European Union (EU), in response to demographic changes and their associated impacts, aims to support Member States in improving care services, including family-based care [1]. Informal care networks, particularly families, constitute the primary source of support for dependent older adults [2], 3]. Nevertheless, providing such care is demanding and requires ongoing adaptation, including the acquisition of skills and the effective application of knowledge and competencies [3].

In Portugal, organizational communication in health aims to systematically disseminate key health-related issues among various information agents in order to promote changes and improvements in population health, increase social awareness of the sustainable use of the healthcare system, and foster citizens’ skills for health promotion, disease prevention, and treatment. In this context, Health Literacy (HL) plays a fundamental role [4].

HL is a key concept for healthcare professionals and is commonly described across three domains: healthcare (HC), disease prevention (DP), and health promotion (HP). The HC domain refers to the ability to access, understand, interpret, and use relevant health information to make informed decisions. DP involves the ability to access, interpret, and critically appraise information related to health risk factors. HP encompasses the ability to understand and interpret health information and to form informed judgments on health-related issues, thereby supporting professional practice in healthcare, particularly in nursing [5], 6].

In the late 1990s, the first formal definitions of HL emerged, expanding the concept beyond the individual dimension to include a social component related to informed decision-making and responsibility for those decisions [7]. This perspective is consistent with the World Health Organization (WHO), which in 1998 defined HL as encompassing cognitive abilities to understand health information, as well as the skills required to perform calculations and interpret complex data in order to make informed decisions [8].

In turn, Nutbeam [9] proposed a conceptual model of HL oriented toward health promotion by healthcare professionals, highlighting the links between individuals’ general literacy and their level of HL.

Between 2000 and 2010, the concept of HL expanded to incorporate dimensions related to access to new information and communication technologies, as well as the ability to locate, evaluate, and use credible information sources. This evolution reflects a dynamic process of adaptation to the challenges posed by the increasing availability of information [10].

According to the WHO, HL functions as a guiding framework for health education, playing a crucial role in informed decision-making, empowerment of individuals and communities, and the promotion of inclusive and equitable access to quality education and knowledge throughout the life course. HL is regarded as an integral component of health promotion, influenced by cultural and contextual factors related to individuals, organizations, and society. In this framework, institutions such as governments, communities, and health services are viewed as key information providers. HL therefore encompasses the availability of reliable and understandable information for all, shaped by social HL resources, regulatory contexts, and the communication media structures (oral, print, broadcast, and digital) within a community [11].

According to Vilhena, improving the various domains of HL contributes, in collaboration with healthcare professionals, to the development of more informed, aware, and autonomous users and citizens in making decisions about their health. Within a biomedical framework, it is essential to promote investment in biopsychosocial dimensions, technical and expert knowledge, and skill development, while also addressing confidence, motivation, and self-efficacy (SE), as well as strengthening health communication [12].

Measuring HL provides a foundation for planning interventions aimed at improving HL itself and population health. It also supports the development of health policies and enables the identification of specific population groups and priority areas requiring improvement across HL subdomains, including access to, understanding of, evaluation of, and application of health-related information. Evidence indicates that adequate levels of HL are directly associated with health outcomes, patterns of healthcare utilization, and healthcare costs [13].

HL promotion has become a public health priority in Portugal [14], 15]. Accordingly, understanding the level of HL and its determinants enables informed decision-making regarding HC, DP, and HP, with the ultimate goal of improving quality of life across the life course. This life-course approach encompasses actions tailored to different life transitions and promotes a comprehensive, society-wide intervention strategy [16].

The WHO [16] has set the challenge of achieving significant and measurable improvements in the lives of older people, their families, and communities by 2030. This objective requires the establishment of partnerships with older adults, policy makers, and stakeholders involved in the development of community-based programs. The intended transformation focuses on four key areas: (i) a strong commitment to older people, families, caregivers, and other relevant actors; (ii) the promotion of integrated, cross-sectoral actions; (iii) the strengthening of collaboration among stakeholders through the sharing of experiences and knowledge; and (iv) the promotion of research, innovation, and data generation to accelerate progress toward these goals.

The 2019–2021 Health Literacy Action Plan highlighted the role of HL during the COVID-19 pandemic, emphasizing the need to promote HL across all implementation contexts. It underscored the importance of improving the quality and accessibility of health information, reaching populations through micro-influencers (e.g., informal caregivers [IC]), enhancing the quality of communication on health-related topics, and promoting HL assessment in specific population groups, including its integration into Primary Health Care information systems. These initiatives should adopt interdisciplinary and multifactorial approaches that address the various domains of HL, with the aim of reducing health inequities [6], 17].

The home became the primary setting of care, and families emerged as the main socio-familial reference contexts, highlighting the central role of family caregivers (FC). The pandemic also brought renewed attention to citizens’ capacity to make health-related decisions, which depends on their ability to seek information and access health care through multiple resources [18].

In the current sociodemographic context, caregivers often assume care responsibilities in transitional situations without prior notice or planning, frequently without a clear understanding of what caregiving entails. Their information-seeking behavior is typically limited to basic issues such as benefits and entitlements, support services, employment conditions, and opportunities for skills development. Caregiver associations acknowledge the need to establish “one-stop shops” that provide centralized access to information on care delivery, caregiving roles, and available support measures. Such initiatives would help caregivers deliver high-quality care while maintaining a productive and healthy life. These associations also emphasize solutions based on information and communication technologies and peer-support initiatives, which should be actively encouraged and supported [19].

Home-based care provided either by citizens themselves or by FC has increasingly emerged as a viable solution for the future of care delivery. However, it is essential to assess whether citizens are adequately prepared to assume this responsibility and whether health professionals are equipped to respond to this evolving care paradigm. For individuals to take an active role in managing their own health, HL represents an emerging public health challenge, characterized by a high level of individual, community, and organizational engagement. HL enables effective self-management of chronic conditions and supports the safe and competent assumption of the FC role [18], 20]. Public authorities and relevant stakeholders should therefore raise awareness of the importance of informal care and systematically collect data on the number, characteristics, needs, and preferences of caregivers through national censuses, surveys, and the development of self-identification tools [19].

To promote self-care - either for oneself or for dependent individuals – it is crucial to provide clear, positive, and assertive information, alongside the development of cognitive, social, and behavioral skills aimed at facilitating behavior change [20], 21]. Communication strategies should be carefully planned and tailored to activate health-protective behaviors, with particular attention to target groups such as FC. Identifying appropriate communication channels and strategic partners is essential to ensure effectiveness and reach [6].

Caregiver training enhances confidence and contributes to the provision of high-quality care over longer periods and under better conditions for both caregivers and care recipients. Preventive approaches that support well-trained and adequately resourced IC have been shown to prevent or delay hospital admissions and reduce the need for long-term institutional care-outcomes. Furthermore, recognizing, developing, and validating the wide range of skills and competencies acquired by FC through their caregiving roles contributes to the sustainability of care systems and to the broader socio-economic balance within the EU [19]. Caregiver organizations also emphasize the need to promote and regulate financial support for caregivers, encourage employment practices that are flexible and compatible with caregiving responsibilities, and involve multiple stakeholders in the development and implementation of legislative and practical measures to support caregivers [19].

Assessing the FC role is of major importance for nurses, as it enables an appreciative perspective and the development of strategies to address problems related to family interaction patterns involving dependent members. The FC role includes supporting the dependent family member (DFM) in self-care arising from illness or disability and encompasses multiple dimensions, such as role-related knowledge, adherence behaviors, role consensus, role conflict, and role overload [22]. Nurses, as key reference professionals within health care systems, play a central role in the education and training of FC [23].

Within the context of Family Health Nursing, supporting the development of SE is particularly relevant. By adopting a patient- and family-centered approach, nursing professionals can improve health outcomes, strengthen family relationships, and promote overall well-being [24].

Specifically, the SE of FC, who provide ongoing support to family members experiencing health vulnerabilities, chronic illness, disability, or functional limitations, is a central component of family health care practice [3], 25].

Caregivers with high SE tend to feel more capable of managing caregiving responsibilities and coping with the stress associated with this role, thereby presenting a lower risk of burnout and psychological distress [3], 26].

According to R. Santos et al., SE is an important indicator in the transition to the FC role, however, it should be complemented by structured intervention programs aimed at promoting this role, particularly with a person-and family-centered focus [27].

F. Santos et al. further demonstrate that family-based interventions are effective in enhancing caregiver SE and confidence, leading to improvements in both the quality of care provided to the DFM and the overall well-being of the FC. Interventions that take into account the caregiver’s trajectory, knowledge, and skills should be strengthened, as higher SE is consistently associated with greater caregiver well-being and quality of life [3].

Family health nurses play a pivotal role in supporting FC and enhancing their SE [26]. FC with higher SE show lower levels of burnout and depressive symptoms, as well as greater satisfaction in performing the caregiving role [28].

In summary, SE emerges as a key factor in assuming the FC role, acting as a mediator of role-adherence behaviors and influencing both the quality of care delivered and the caregiver’s psychological well-being [3], 28].

Consequently, the assessment of the FC role is fundamental, and nurses must be equipped with appropriate conceptual frameworks, validated assessment instruments, and the ability to adapt their clinical practice based on assessment outcomes [29].

Within the caregiver role, adherence behaviors constitute a key subdimension, focusing on the effective performance of caregiving tasks. These behaviors include stimulating the DFM’s independence; promoting appropriate personal hygiene; ensuring suitable clothing and adequate nutritional intake; facilitating the acquisition and use of adaptive equipment for toileting; promoting healthy sleep-rest patterns, recreational activities, and appropriate exercise; and supporting the DFM in self-monitoring activities [22].

Considering the limitations and disabilities of the DFM, it is essential to assess the FC’s level of role-related knowledge in performing activities of daily living, as well as the degree of adherence to the FC role. This includes evaluating both the caregiver’s commitment and the effective execution of caregiving tasks for the DFM [30].

In summary, SE plays a central role in adherence behaviors related to the FC role. Strategies aimed at enhancing SE contribute to improved adherence to caregiving responsibilities and promote the well-being of both the caregiver and the care recipient. Assessing the FC’s SE in behaviors related to adhering to the role of FC, is therefore particularly relevant, as it is closely associated with a sense of mastery in responding to the DFM’s needs.

It is within the scope of this problem that this study arises, which seeks to analyze the relationship between the FC’s HL and their perception of SE in the behaviors of adherence to the FC role.

The importance of this study is based on the relationship between adequate levels of HL and better health outcomes, and because HL supports the safe and competent assumption of the role of FC and the improvement of their SE, which in turn influences the effective execution of tasks and the quality of care provided by FC. This importance can also be based on the contribution that this study may have to achieving the challenge set by the WHO, to achieve significant improvements in the lives of older people and their families by 2030.

To achieve these goals, we started from the hypothesis that there is a relationship between the level of HL of FC and their perception of SE in adherence behaviors to the role of FC, and from the initial question of what is the relationship between the level of HL of FC´s and their perception of SE in adherence behaviors to the role of FC.

## Methods

### Study design and participants

We developed an observational, descriptive-correlational, cross-sectional study with a quantitative approach that analyzed the characteristics of a population and established a relationship between variables [31].

We defined the following inclusion criteria for this study: i) FC of users registered at the Family Health Unit (FHU) in the northern interior of Portugal, with the Dependent Program open at SClínico, in the period between January and February 2024; ii) FC of dependent users not residing in residential structures for the elderly and in foster families; iii) FC of dependent users registered at the FHU in the context of this study who could be contacted and located during the data collection period. After applying the same criteria, the estimated size of this population was 131 FC of users registered at a FHU in the northern interior of Portugal, with a Dependent Program open at SClínico Nursing Profile, in the period between January and February 2024.

From a population of 131 FC, we applied the Araldi [32] formula n=[(N × n_0_)/(N + n_0_)], using a sampling error of 5 % and a confidence interval of 95 %. The n is the minimum sample size, N is the population size, and n_0_ corresponds to the first approximation to the sample size, calculated from the formula 1/(E_0_)^2^, where E_0_=0.05. The minimum sample size is calculated as n=(131 × 400)/(131 + 400)=99 FC. To this minimum sample size (99 FC) 12 more FC were added to guarantee this minimum, which totaled 111 FC. This sample allowed us to have a minimum number of FC classified into each of the SE categories (Incompetent and Competent) to perform the Mann–Whitney (MW) test.

The sample studied excluded the elements of the population that met the following exclusion criteria: i) FC who changed FHU during the data collection period; ii) FC of users who died during the data collection period; iii) FC who did not respond to at least 14 items on the HL scale. As none of the FC changed FHU, nor died, nor were excluded for not having completed at least 14 items of the HL scale during the data collection period, the sample consisted of 111 FC.

This is a non-probabilistic convenience sample, or accidental sample, whose constituent elements of the sample did not have the same probability of being part of it.

### Data collection instrument

The data collection instrument used in this study was a form, which was organized into five parts: the first part was intended to collect sociodemographic data; the second included questions to characterize the FC family; the third part consisted of the European Health Literacy Survey scale (short version HLS-EU-PT-Q16), to assess the level of HL of the sample; the fourth part included a set of questions to assess the level of SE in the FC role adherence behaviors of our sample; the fifth part intended to assess the level of dependency of the person cared for by applying the Barthel Index, in a community context. This manuscript covers only the data collected in the first, third and fourth parts.

The third part involves the application of the HLS-EU-PT-Q16, which measures the level of HL, based on the caregiver’s self-perception in the performance of a set of activities/tasks in the health domain, consisting of 16 questions/items in three assessment domains: access to HC through seven items, DP through five items and HP through four items. The questionnaire aims to understand the respondents’ ability to Access, Understand, Appreciate and Apply information relevant to health in the three domains mentioned. The respondent assigns, based on self-perception, a level of difficulty in performing tasks, on a scale of 0–4 points and the respondent has to answer at least 14 of the 16 questions. For effective comparison of the subdomains, the indices were standardized in the general HL index (HLI), according to a metric scale that varies between 0 and 50. The general HLI (G-HLI 16) was calculated as follows: G-HLI 16=(mean − 1) × (50/3). Where the “mean” is the mean of all the participating indices for all. For the different participation indices, four HL levels were defined: inadequate (0–25), problematic (25.1–33), sufficient (33.1–42) and excellent (42.1–50). The response “don’t know/no answer” was recoded as missing. Cronbach’s alpha coefficient for the internal consistency of the HLS-EU-PT-Q16 was as follows: 0.783 for the HC domain; 0.724 for the DP domain; 0.703 for the HP domain; and 0.890 for the General HL, Cronbach’s alpha ranges from Good to Very Good [33].

The choice of this scale, a reduced version of the HLS-EU-PT scale, was related to its validation in the Portuguese population by Pedro et al., due to the fact that it presents fewer questions than the scale from which it was derived, the HLS-EU-Q47 (most commonly used in Portugal) and is more reliable than the HLS-EU-PT-Q6, making the instrument adaptable to the demanding demands of the FC and take into account the fact that the sample data collection period is short [33].

The fourth part involves the application of a questionnaire to assess the level of SE in the FC role adherence behaviors of our sample. To assess the level of SE in the FC role adherence behaviors, we used nine questions constructed by Figueiredo [22], which constitute a subdimension of the Dynamic Model of Family Assessment and Intervention, available in SClínico, an information system accessible in the context of the FHU under study. The questions allow us to verify the effective development of nine tasks in the care of the DFM [22]. For each adherence behavior, the FC defines its level of SE through five response options: Level 0 “Does not know or Does not answer”, Level 1 “Incompetent”, Level 2 “Somewhat competent”, Level 3 “Moderately competent” and Level 4 “Very competent”. We associated the first two lower levels (Level 1 and Level 2) and the two higher levels (Level 3 and Level 4), then called, respectively, “Incompetent” and “Competent”, in order to allow the execution of statistical tests. We do not intend to measure the general perception of SE in the development of the FC role, as adherence behaviors do not reflect all the tasks performed by the FC. Thus, we intend to understand SE in the performance of each of the nine tasks separately.

### Ethical considerations

In scientific research involving ethical and moral sensitivities and the participation of human beings, we must adhere to a set of principles, namely, respect for free and informed consent, confidentiality, justice and equity. Given that these are highly vulnerable groups, such as dependent people and, more often, elderly people and their caregivers, we emphasize respect for the privacy of the person and their family, moral, physical and human integrity, obtaining only the personal data essential for carrying out the study.

In order to respect ethical principles, authorization to carry out the study was requested from the FHU Coordinator, including the endorsement of the Person Responsible for Access to Information (RAI) and the Executive Director of the Health Centers Group (ACeS), as well as the opinion of the Ethics Committee of the Northern Regional Health Administration (ARS Norte), which issued a favorable opinion (Opinion CE/2024/9 of 11-01-2024). Informed Consent was requested from all participants, who authorized participation in the research, in the data collection process and were informed about anonymity, confidentiality, the time of completion and that they could withdraw at any time. This study was conducted in accordance with the Declaration of Helsinki (as revised in 2013).

### Data collection procedure

Data collection was carried out through a questionnaire interview (form) conducted by telephone by the researchers, from January 12 to February 26, 2024, during the internship at FHU, the context of this study. The researchers began the data collection process by identifying themselves, presenting the study’s theme and objectives, and guaranteeing the confidentiality of the data, which would be used exclusively for academic purposes, as well as the possibility for participants to withdraw from the study at any time without any personal penalty. Next, the first question asked of FC, who were part of the population, was whether they consented to participate in the study. If they did not consent, the process was immediately interrupted. The same procedure was always followed. This collected data was recorded in the data collection instrument by the researchers.

### Data analysis

For data processing and analysis, a database was constructed in the statistical software Statistical Package for Social Sciences (SPSS version 29.0), into which they were entered. In data processing, descriptive and inferential statistics were used. In terms of descriptive statistics, the calculation of absolute and relative frequencies was requested for all variables under study and of measures of central tendency and dispersion for variables at the ratio measurement level (General HL and of each of its subdomains). In terms of inferential statistics, the associations between the General HL variable and each of the categories (Incompetent and Competent) of perceived SE in adhering to the role of FC were evaluated, namely: Encourage DFM Independence, Promote adequate hygiene for DFM, Promote use of appropriate clothing for DFM, Promote adequate nutritional intake for DFM, Acquire adaptive equipment for toilet use, Promote adequate sleep and rest behaviors for DFM, Promote adequate recreational activities for DFM, Promote adequate exercise pattern for DFM, and Assist DFM in self-monitoring, using the MW statistical test. This statistical test was performed to answer the study’s objective of analyzing the relationship between the level of FC´s HL and their perception of SE in behaviors related to adhering to the role of FC. This non-parametric test was chosen because the assumptions for applying parametric tests (normality and homogeneity of variances) were not met. The significance level used was 5 % [34].

## Results

Of the total FC sample (n=111), the majority were female (82.9 %), belonged to the age group of 45–64 years (52.3 %), had a marital status of married/*de facto* union (64.0 %), regarding Socioeconomic Level, the largest group belonged to the middle class (33.3 %), the majority took care of their mother/father (55.9 %) and were inactive in their work situation (56.8 %).

The HC dimension showed the highest percentages of responses in the “Easy” and “Very easy” options. In item 7 (processing level “Use”), 82.9 % and 6.3 % were recorded, respectively. This is followed by the DP dimension, which in item 10 (level “Understand”), obtained 82 % “Easy” responses and 7.2 % “Very easy”. Finally, the HP dimension stood out in item 16 (level “Evaluate”). It recorded 87.4 % “Easy” responses and 4.5 % “Very easy”. These three items were the ones that contributed most to a high level of General HL (Table 1).

**Table 1:** Percentage of response options for the health literacy scale of the sample to the 16 items (%).

HLS items – EU-PT Q16	Domain/processing level	Very difficult	Difficult	Easy	Very easy	D/K D/A
1. “Looking for information on treatments for conditions that concern you?”	HC To access	5.4	45.0	47.7	1.8	0.0
2. “Want to know more about where to get expert help when you’re sick?”	HC To access	5.4	43.2	49.5	1.8	0.0
3. “Understand what your doctor tells you?”	HC To understand	0.0	18.0	73.9	8.1	0.0
4. “Understand your doctor or pharmacist’s instructions about taking a prescribed medication?”	HC To understand	0.9	9.9	82.0	7.2	0.0
5. “Assess when you might need a second opinion from another doctor?”	HC To assess	1.8	29.7	61.3	4.5	2.7
6. “Use information your doctor gives you to make decisions about your illness?”	HC To use	0.9	24.3	71.2	3.6	0.0
7. “Follow your doctor or pharmacist’s instructions?”	HC To use	0.9	9.9	82.9	6.3	0.0
8. “Finding information to deal with mental health issues like stress or depression?”	DP To access	4.5	48.6	45.9	0.9	0.0
9. “Understand health warnings regarding behaviors such as smoking, talking about physical activity and excessive alcohol consumption?”	DP To understand	0.0	11.7	82.0	6.3	0.0
10. “Understand why you need to do screenings?”	DP To understand	0.0	10.8	82.0	7.2	0.0
11. “Assess whether information in the media about health risks is reliable?”	DP To assess	2.7	47.7	47.7	1.8	0.0
12. “Deciding how to protect yourself from the disease based on media information?”	DP To use	4.5	46.8	47.7	0.9	0.0
13. “Want to learn more about activities that are good for your mental well-being?”	HP To access	2.7	25.2	67.6	3.6	0.9
14. “Understanding health advice from family or friends?”	HP To understand	0.9	18.8	74.8	4.5	0.9
15. “Understand the information in the media about how to stay healthier?”	HP To understand	0.9	26.1	70.3	2.7	0.0
16. “Assess which daily behaviors are related to your health?”	HP To assess	1.8	6.3	87.4	4.5	0.0

DP, disease prevention; HC, health care; HLS-EU-PT Q16, health literacy scale, with 6 items; HP, health promotion; DK/DA, don’t know or don’t answer.

Regarding HL indexes, the highest average score was observed in the HP subdomain (30.03 %). This subdomain also presented the highest standard deviation (7.15 points) (Table 2).

**Table 2:** Measures of central tendency and dispersion of the general HLI and its subdomains in the total sample (n=111).

Variables	Mean	Median	Mode	SD	Min.	Max.
HL general index	28.78	30.21	33.33	6.48	11.46	50.0
HC domain index	28.91	30.95	33.33	7.14	11.90	50.0
DP domain index	27.60	30.00	33.33	6.65	10.00	50.0
HP domain index	30.03	33.33	33.33	7.15	0.00	50.0

DP, disease prevention; HC, health care; HL, health literacy; HP, health promotion; Min., minimum; Max., maximum; n, total sample; SD, standard deviation.

Regarding the categorization of HL levels, in the General HL, the largest group presented a problematic level (47.7 %). In the HC subdomain, the most frequent level was also problematic (37.8 %). In the DP subdomain, the adequate and problematic levels had the same percentage (34.2 %). In the HP subdomain, the largest group presented a sufficient level (52.3 %) (Table 3).

**Table 3:** Categorization of HLIs in the general index and its subdomains (n=111).

Variables	Inadequate 0–25.0 points	Problematic 25.1–33.0 points	Sufficient 33.1–42.0 points	Excellent 42.1–50.0 points
HL general	25.2	47.7	23.4	3.6
HL health care	24.3	37.8	32.4	5.4
HL disease prevention	34.2	34.2	29.7	1.8
HL health prevention	23.4	20.7	52.3	3.6

HL, health literacy.

The variable level of SE in behaviors related to adherence to the role of FC was recoded into two groups, with the groups “Incompetent” and “Somewhat competent” and the groups “Moderately competent” and “Very competent” associated in the new category “Incompetent” and “Competent”, respectively. Thus, the behaviors in which the sample was considered most “Incompetent” were “Promoting recreational activities to the DFM”, “Promoting an appropriate exercise pattern to the DFM” and “Encouraging the DFM’s independence”, with 76.6 , 74.8 and 70.3 %, respectively. In contrast, the behaviors in which the sample was considered most “Competent” were “Assisting the DFM in self-monitoring”, “Promoting adequate nutritional intake to the DFM” and “Promoting the use of appropriate clothing to the DFM”, with percentages of 78.4 , 75.7 and 73.9 %, respectively (Table 4).

**Table 4:** Perception of SE in the behaviors of adherence to the recoded FC role.

FC role adherence behaviors	SE Perception, %	
	Incompetent	Competent
AB 1 – encourage DFM independence	70.3	28.8
AB 2 – promote proper hygiene of the DFM	39.6	60.4
AB 3 – promote the use of appropriate clothing by the DFM	26.1	73.9
AB 4 – promote adequate nutritional intake by the DFM	24.3	75.7
AB 5 – acquire adaptive equipment for the DFM’s use of the toilet	44.1	55.0
AB 6 – promote sleep-rest behavior of the DFM	25.2	74.8
AB 7 – promote recreational activities	76.6	22.5
AB 8 – promote adequate exercise pattern	74.8	24.3
AB 9 – assist the DFM in self-monitoring	21.6	78.4

AB, adherence behavior; FC, family caregiver; DFM, dependent family member; SE, self-efficacy.

The mean rank of the general HLI categories differed significantly in the FC role adherence behaviors “Encouraging the DFM’s independence” (MW: p=0.033), “Promoting the use of appropriate clothing for the DFM” (MW: p=0.001), and “Assisting the DFM in their self-monitoring” (MW: p=0.000).

Thus, the mean rank of the HLI in the SE categories in the behavior “Encouraging the DFM’s independence” was higher in competent FC´s and lower in incompetent FC´s (65.55 points >51.38 points), that is, FC with a higher level of HL had higher SE in this behavior. The mean rank of the general HLI in the categories of SE in the behavior “Promote the use of appropriate clothing for the DFM” was also higher in competent FC´s and lower in incompetent FC´s (61.88 points > 39.38 points), that is, FC with higher HLI had a higher SE in this behavior. The same occurred regarding the mean rank of the general HLI in the categories of SE perception in the behavior “Assist the DFM in his/her self-surveillance”, which was higher in competent FC´s and lower in incompetent FC´s (65.52 points > 35.38 points), that is, FCs with a higher level of HL had a higher SE in this behavior (Table 5).

**Table 5:** Results of statistical tests between the health literacy index variable and the perception of self-efficacy variable in the behaviors of adherence to the role of Family Caregiver (n=111).

Variables		Af	Mean rank	df	Test value	p-Value
HLI* item 1	Incompetent Competent	78 32	51.38 65.55	__	MW=926.50	0.033
HLI* item 2	Incompetent Competent	44 67	52.86 58.06	__	MW=1,336.00	0.403
HLI* item 3	Incompetent Competent	29 82	39.38 61.88	__	MW=707.00	0.001
HLI* item 4	Incompetent Competent	27 84	54.65 56.43	__	MW=1,097.50	0.801
HLI* item 5	Incompetent Competent	49 61	52.20 58.15	__	MW=1,333.00	0.329
HLI* item 6	Incompetent Competent	28 83	50.05 58.01	__	MW=995.50	0.256
HLI* item 7	Incompetent Competent	85 25	57.81 47.64	__	MW=866.000	0.159
HLI* item 8	Incompetent Competent	83 27	53.71 61.00	__	MW=972.000	0.300
HLI* item 9	Incompetent Competent	24 87	35.38 65.52	__	MW=477.000	0.000

Af, absolute frequency; df, degree of freedom; HLI, health literacy index; Item 1, encourage DFM independence; Item 2, promote adequate hygiene for DFM; Item 3, promote use of appropriate clothing for DFM; Item 4, promote adequate nutritional intake for DFM; Item 5, acquire adaptive equipment for toilet use; Item 6, promote adequate sleep and rest behaviors for DFM; Item 7, promote adequate recreational activities for DFM; Item 8, promote adequate exercise pattern for DFM; Item 9, assist DFM in self-monitoring; MW, Mann–Whitney; KW, Kruskal–Walls; p-level of significance.

## Discussion

In the present study, we found that there is a statistically significant dependency relationship between the General HL and the categories of SE (Incompetent and Competent) in the behavior of adherence to the role of FC “Encouraging the independence of the DFM”, “Promoting the use of appropriate clothing for the DFM” and “Assisting the DFM in their self-monitoring”, with FC with higher Health Literacy always perceiving themselves as more competent in performing these three behaviors.

Regarding the distribution of responses to the HLS-EU-PT-Q16, it is possible to verify a predominance of easy and very easy responses for all questions, except items 8, 11 and 12. These results are similar to the results obtained in the study by Veladas [35], with a sample of 204 health professionals and students, from the Lisbon and Tagus Valley area (Portugal), with the objective of analyzing the level of HL and HL in Oral Health, through the HLS-EU-PT-Q16 Scale, in which except item 11, all items obtained a positive response.

In turn, in the study by Vaz [36], with a sample of 212 individuals, with the objective of evaluating the HL of 106 formal caregivers and 106 Informal Caregivers, from the north coast of Portugal by applying the HLS-EU-PT with 47 items, item 11 “assess whether the information in the media about health risks is trustworthy?” (48.1 %) and item 8 “… find information to deal with mental health problems, such as stress or depression?”, stood out as being difficult for the largest group in the sample, which highlighted weaknesses in the knowledge of FC in areas similar to those of the present study.

Considering the present study and the studies carried out in the Lisbon and Tagus Valley area [35] and in the North of Portugal [36], the need to invest in the prevention of mental illness is evident, and the difficulty of preventing the disease through the information provided in the media is highlighted.

Regarding the categorization of general HLI levels, the largest group presented a problematic level (47.7 %), data that are in line with the study by Escoval et al. [37] who applied the HLS-EU-PT-Q16 to 760 Portuguese FC and the one carried out in the Lisbon and Tagus Valley Region [35], in which the largest group in the sample fell into the problematic HL level, respectively, 40.9 and 45.1 % of the sample.

This result is similar to that obtained in the study carried out in the North Coast of Portugal [36], although there is a difference in the assessment scale used, HLS-EU-PT (47 items), the majority of elderly IC (51.4 %) have problematic general HLI, with low levels of excellent in both studies [37]. The results are in line with those obtained by Pedro et al. [38], who validated the HLS-EU-PT-Q16 scale with 1,000 individual’s representative of the Portuguese population and the results of the Health Literacy Survey EU 2014, where Portugal is the country with the lowest excellent HL level and a problematic level above the European average [4], 33], 38].

Comparing the data obtained in the present study with the data contained in the Health Literacy Action Plan Report 2019–2021, which assessed the HL of the Portuguese population by applying the HLS-Q12 to 813 individuals, they are distinct regarding the general HL given that the majority had a sufficient level of HL [4].

The results obtained may be due to the positive impact of strategic policies at national level to increase the level of HL of the populations, including digital HL, the national implementation of priority health promotion programs and the implementation of programs such as the support program for health promotion and education, with multi-ministerial involvement.

Regarding SE in the behaviors of adherence to the FC´s role, a study carried out in Paços de Ferreira (Portugal) [25], with 2,126 families, made a descriptive analysis regarding the encouragement of DFM by the FC, in the domain of self-care and evaluated the perception of SE of 1,279 FC, for the various self-care. Comparing the results of the present study with those obtained by this study [25] we can see that the FC considered themselves competent in the performance of self-care. In the present study, for almost all the behaviors of adherence to the FC role, the majority considered themselves competent, except for the behaviors: “Encouraging the independence of the DFM”, “Promoting recreational activities appropriate to the DFM” and “Promoting an exercise pattern appropriate to the DFM”, in which the majority perceived themselves as Very incompetent. The differences found, small variations depending on the domain of self-care in question, may derive from the degree of demand of the care to be performed, allusion to the lack of time, FC overload, in the home context, which will imply a pattern of instrumental and substitutive care for the dependent and greater focus on activities of promotion and maintenance of life.

In the quantitative and correlational study carried out in a hospital in Concepción (Chile) [39] that aimed to understand the SE of 97 FC people in critical condition and relate it to the biopsychosocial characteristics and morbidity indicators, high perceptions of SE for self-care (64 %) were reported, similar to the present study for most of the behaviors of adherence to the FC role.

Regarding the relationship between the FC’s HL level and SE in the FC role adherence behaviors “Encouraging the DFM’s independence”, “Promoting the use of appropriate clothing for the DFM” and “Assisting the DFM in their self-monitoring”, FC with higher HL had higher SE in these behaviors. According to the study contained in the Health Literacy Action Plan Report 2019–2021 [37], the FC’s HL level is directly related to the perception of competence to provide care. Thus, FC with higher HL levels have a greater perception of competence in providing care, which is in line with the results obtained for the three behaviors highlighted in the previous paragraph.

These results highlight that higher levels of HL promote autonomy, improve the quality of life of citizens and the quality of care provided. According to Almeida [5], the increase in the level of HL of citizens makes them responsible and proactive in maintaining healthy families and communities and better skills to use basic health information and benefit the context. The level of HL influences conceptions and beliefs about health. The way people cope with illness is related to the resources, knowledge, attitude, beliefs and perceptions they have about health.

Furthermore, according to the study included in the Health Literacy Action Plan Report 2019–2021 [37], by applying the HLS-EU-PT-Q16, low levels of HL are associated with poorer health care provision by FC, worse health outcomes for care recipients, and greater burden on caregivers.

The FC profile of this sample is composed of a middle-aged, married female, daughter of a dependent, and inactive in terms of employment. The HL level is mostly problematic, which, combined with inadequate, accounts for almost three-quarters of limited HL. We can also conclude that the FC in this sample consider themselves to be mostly competent in almost all behaviors of adherence to the FC role.

HL is partially related to SE in the behaviors of adherence to the FC role, since only three of the nine behaviors showed statistically significant differences, always with the FC who considered themselves competent having greater HLI.

### Strengths and limitations of the study

In terms of the strengths of this study, the following stand out: the use of a topic that has been little studied and the potential application of the results to improve professional practices; the use of a validated and reliable scale; the high homogeneity of the sample, ensured through inclusion and exclusion criteria; and the use of appropriate statistical analysis aligned with the study’s objective.

As the main limitations of this study, we highlight the fact that it is a non-probabilistic sample, for convenience, which may have caused deficiencies in the representativeness of the population, which weakens the statistical inference of the sample for the population. Furthermore, it is a cross-sectional study, in which it is not possible to establish cause-effect relationships.

This study may have implications for the professional practice of researchers and the multidisciplinary health team, specifically the Nursing team, in the context where the study was carried out; with regard to HL and SE in the behaviors of adherence to the FC role, since it constitutes a diagnosis of the situation, paving the way for a better response to the needs identified by caregivers, improving the performance of care for dependent family members and promoting the quality of life of dependents and the FC themselves.

## Conclusions

This study revealed a positive association between the level of HL and the perception of SE in the performance of the role of FC, where higher levels of HL may be decisive in promoting autonomy, the quality of care provided, and improving the quality of life of FC and DFM, which is consistent with studies developed by several authors. Improving the HL level of FC could increase the quality of their performance.
